# Genomic and molecular characterization of a novel quorum sensing molecule in *Bacillus licheniformis*

**DOI:** 10.1186/s13568-017-0381-6

**Published:** 2017-04-08

**Authors:** Elham Esmaeilishirazifard, Daniela De Vizio, Sterghios A. Moschos, Tajalli Keshavarz

**Affiliations:** 1 Department of Life Sciences, Faculty of Science and Technology, University of Westminster, London, UK; 2 GlaxoSmithKline, Worthing, West Sussex, UK; 3 Department of Biomedical Sciences, Faculty of Science and Technology, University of Westminster, London, UK; 4 Department of Applied Sciences, Faculty of Health and Life Sciences, Northumbria University, Newcastle, UK

**Keywords:** Quorum sensing, Pheromone, *Bacillus*, Gene cloning, Peptide purification, Genome sequencing

## Abstract

Quorum sensing molecules (QSMs) are involved in the regulation of complicated processes helping bacterial populations respond to changes in their cell-density. Although the QS gene cluster (*comQXPA*) has been identified in the genome sequence of some bacilli, the QS system *B. licheniformis* has not been investigated in detail, and its QSM (ComX pheromone) has not been identified. Given the importance of this antagonistic bacterium as an industrial workhorse, this study was aimed to elucidate *B. licheniformis* NCIMB-8874 QS. The results obtained from bioinformatics studies on the whole genome sequence of this strain confirmed the presence of essential quorum sensing-related genes. Although polymorphism was verified in three proteins of this cluster, ComQ, precursor-ComX and ComP, the transcription factor ComA was confirmed as the most conserved protein. The cell–cell communication of *B. licheniformis* NCIMB-8874 was investigated through further elucidation of the ComX pheromone as 13-amino acid peptide. The peptide sequence of the pheromone has been described through biochemical characterisation.

**Electronic supplementary material:**

The online version of this article (doi:10.1186/s13568-017-0381-6) contains supplementary material, which is available to authorized users.

## Introduction

The first evidence of microbial cell–cell communication was reported by Tomasz and Beiser in 1965, when they suggested that a hormone-like extracellular product regulated competence in *Streptococcus pneumoniae* (Tomasz and Beiser 1965). Later on researchers found that the product was a peptide acting as a common signal in cell–cell communication amongst Gram-positive bacteria (Dunny and Leonard 1997). However, organized responses in a microbial colony were officially reported in the luminous marine bacterium.

*Aliivibrio fischeri* in its symbiotic relationship with the Hawaiian squid, *Euprymna scolopes*. Bioluminescence was triggered and controlled by one or more signalling molecules accumulating in the extracellular environment of *A. fischeri* as their cell density increased and reached a critical number (quorum). Signal molecules implicated in cell–cell communication are known as auto-inducers and/or QSM and their function is to regulate gene expression in other cells of the community to control bacterial responses (Nealson et al. 1970).

In Gram negative bacteria the QSMs are diffusible, they use low molecular weight hydrophobic signal molecules. Gram-positive bacteria employ unmodified or post-translationally modified peptides as well as γ-butyrolactone. In the cytoplasm, these peptides are produced as precursors of the QSM and then cleaved, modified and exported. Once in the extracellular environment, the peptides are detected via two-component systems (Kleerebezem et al. 1997). In Gram-positive bacteria, the QSMs are secreted to the extracellular milieu and then recognised by receptors which transport the signal across the cell membrane to initiate the target gene transcription (Waters and Bassler 2005). Studies have shown that QS in *Bacillus* species is mediated by small peptides that control competence (for DNA uptake), sporulation and the production of certain secondary metabolites in a cell-density dependent fashion (Bassler and Miller 2013). A competence pheromone in *B. subtilis* was first described genetically in *B. subtilis* subsp. *subtilis* 168 (Magnuson et al. 1994). In this bacterium, cell–cell communication is regulated through the *comQXPA* locus. The products of this system are the ComX pheromone and the two-component transduction system ComP and ComA which regulate the occurrence of natural competence in this bacterium (Weinrauch et al. 1990; Dubnau et al. 1994). The system is activated by accumulation of the ComX pheromone in the extracellular milieu (Magnuson et al. 1994).

Studies by Ansaldi and co-workers confirmed that the *comQXPA* gene cluster plays an essential role in the regulation of competence development in the *B. subtilis* QS mechanism (Ansaldi et al. 2002). This gene cluster is present in bacilli with close genomic relationship to *B. subtilis*, a group within which *B. licheniformis* is reported to belong (Magnuson et al. 1994). De Vizio identified that a *B. licheniformis* NCIMB 8874 cell–cell communication operates analogously to the *comQXPA*-controlled pathway of *B. subtilis* (De Vizio 2011). The products of this system are the ComX pheromone and the two-component transduction system ComP and ComA. ComQ is the only dedicated protein required for the processing of active pheromone (Magnuson et al. 1994).

Although QS is well established in *B. subtilis*, further investigations of the cell–cell communication and signalling molecules in *B. licheniformis* were required as the biochemistry of the relevant QSMs remained unexplored; importantly, such work would help explore potential bio-inhibitory activities relevant to industrial applications such as production of proteases, amylases and specialty chemicals (Schallmey et al. 2004) as well as several antimicrobial compounds, such as bacitracin (Johnson et al. 1945) and the surfactin-resembling lichenysin (Yakimov et al. 1995).

Some researchers have focused on the production of the pheromone as a post-translationally modified peptide which requires processing of the precursor to 10 amino acids, modification of the tryptophan residue and export from the cell by ComQ (Lazazzera et al. 1999). It has also been confirmed that the pheromone was formed by isoprenylation of an inactive precursor peptide (Schneider et al. 2002). Okada and colleagues identified the pheromone structure of *B. subtilis* for the first time and the structure of the resulting 6-amino-acid peptide product as a QSM (Okada et al. 2005). With the *B. licheniformis* genome sequence in hand cloning, expression and purification methods developed for *B. subtilis* (Okada et al. 2005) were adopted for the study of the *B. licheniformis* QS system with special emphasis on its signalling molecule, the ComX pheromone.

In the present work, the genomic studies have focused on the extent of polymorphism presented in the amino acid sequences of proteins involved in the *B. licheniformis* QS system. Besides, the QS study on the ComX pheromone carried out by investigating the putative QS genes (comQX) of *B. licheniformis* NCIMB 8874 and over-expressing comQX genes using gene cloning techniques. It led to detect and identify the novel pheromone peptide using available genomic information (using next generation sequencing platform) on *B. licheniformis* QS genes. The ComX pheromone was purified using biochemical techniques on a recombinant *E. coli* culture, constructed for over-production of the pheromone in the supernatant.

## Materials and methods

### Whole genome sequencing analysis of *B. licheniformis* NCIMB 8874

The genome sequences of *B. licheniformis* NCIMB 8874 were sequenced and determined for the first time on the Ion Torrent Personal Genome Machine (PGM) (Life Technologies, Thermo Fisher Scientific, UK) at Genomic Services, University of Westminster. Following the first stage of sequencing procedure, library construction, the template was prepared through emulsion PCR automated system and then run on the PGM to accomplish the sequencing process (no. of reads was 1,624,672; no. of generated contigs was 168 and achieved overall depth of coverage was 59×). These sequence data have been submitted to the GenBank data bases under the accession number MBGK01000000. Details of data submission can be found at GenBank: http://www.ncbi.nlm.nih.gov.

Assembled DNA sequences data in FASTA format was obtained from the Ion Reporter 5.0 software. The assembled sequence was annotated through IonGap Annotation Service (http://iongap.hpc.iter.es/), an integrated Genome Analysis Platform for Ion Torrent sequence data. The phylogenetic analysis was carried out by aligning amino acid sequences of comQXPA cluster from the strain with homologous proteins from other Bacilli which obtained from NCBI nucleotide/protein database using “Clustal Omega” as a multiple sequence alignment program (for details of clustering method please refer to http://www.ebi.ac.uk/Tools/msa/clustalo/).

### Strains, media and general methods

The QSM studies were performed on *B. licheniformis* NCIMB 8874. The reporter strain *B. subtilis* JRL293 [amyE: (*srfA*-*lacZ*, cat), trp, phe] was used for pheromone bioassay. Both strains were available in the Culture Collection of the University of Westminster, London, UK. Lysogeny broth (LB) and LB agar (LBA) (Sigma) were used for the maintenance of *B. licheniformis* NCIMB 8874. Maintenance medium for *B. subtilis* JRL293 was supplemented with chloramphenicol (Sigma) (5 μgml^−1^).

The expression strain [*E. coli* BL21 (DE3)] and *E. coli* TOP10 were used for cloning/transformations and were selected on LBA supplemented with ampicilin (100 µgml^−1^). *E. coli* BL21 ComX producer strain was cultivated in M9 minimal salts solution (sigma). The medium was supplemented with a mix of filter-sterilised amino acids (leucine, phenylalanine, histidine, serine, 40 µgml^−1^ each; glutamine, 400 µgml^−1^), and ampicillin (100 µgml^−1^). According to the manufacturer instruction, additional supplementation of filter-sterilised 20% (w/v) glucose, 1 M magnesium sulfate and 1 M calcium chloride was required in order to complete M9 minimal medium preparation. Filter sterilization was carried out through a 0.22 µm filter (Millipore).

### Plasmid construction for gene cloning

Plasmid allowing the overproduction of ComQ and ComX proteins in *E. coli* was derived from the pET-22b(+) vector. *comQ* and *comX* were PCR amplified from chromosomal DNA with the custom primer set (*comQ*-Forward/*comX*-Reverse). Forward primer (ACGTCATATGAATCATTTTATAGACGTTGAGATTCC) hybridized to a sequence upstream of *comQ* contained a *Nde*I site while downstream *comX* was amplified by reverse primer (ACGTGGATCCTTATTTGAACCATAAATTAGGGTAAG) containing a *Bam*HI site. The annealing temperature was 53 °C and the expected PCR product fragment was 1070 bp. Primers were custom prepared by Invitrogen (Thermo Fisher Scientific).

After cleavage with *Nde*I and *Bam*HI, DNA fragments were cloned into the pET-22b(+) vector cut with the same enzymes. The recombinant plasmid were transformed into *E. coli* TOP10 and then into *E. coli* BL21 (DE3) as a host to express the ComX pheromone. All cloned fragments in both transformation steps were sent for sequencing using the T7 primers (Novagen) to determine the accuracy of their sequence.

### Pheromone overproduction and purification

*Escherichia coli* BL21 ComX producer strain was grown overnight in the completed M9 minimal salts medium described earlier. At stationary phase, this pre-culture (20 ml) was added to 1980 ml of the supplemented M9 medium to make 2 l bacterial culture (5 flasks in total to prepare 10 l culture) and then incubated at 37 °C and 110 rpm for 8 h. *comQX* gene expression was induced with 0.5 mM Isopropyl β-d-1-thiogalactopyranoside (IPTG) at 37 °C and 110 rpm overnight. The culture broth (10 l) was centrifuged for 10 min at 8000*g*. The supernatant was filtered through a 0.22 µm vacuum filtration unit Corning (Sigma). Reverse-phase Chromatography method (RP + C18) was performed for the initial purification and concentration of the filtered supernatant using Diaion HP-20 resin. The eluted solution was collected using absolute acetonitrile. It was then concentrated and dried through rotary evaporator and freeze dryer respectively.

The dried extract from reverse-phase chromatography was analysed through HPLC for the presence of the ComX pheromone. The column was C18 (Thermo Fisher Scientific, 5 μm × 4.6 × 150 mm) with Acclaim 120, C18 5 μm Guard Cartridges (4.6 × 10 mm) on a Dionex ICS-5000 HPLC instrument (Thermo Fisher Scientific). The dried extract was re-dissolved in 200 µl acetic acid, 600 µl acetonitrile and 1200 µl deionised water (1:3:6) to prepare a solution of 25 mgml^−1^. Two different sequences of amino acids were synthesised as standard (standard 1 and standard 2) which obtained from Pepceuticals Ltd. (Leicestershire, UK). These standard samples were used for pheromone quantification and also to confirm the retention time. To find the standard pheromone sequences for the HPLC run, the whole genome sequence of *B. licheniformis* NCIMB 8874 and the sequence of the recombinant plasmid (carrying *comQX*) were studied. The potential pheromone sequence as standard 1 was obtained from the whole genome sequence of *B. licheniformis* NCIMB 8874 and the corresponding amino acid sequences were identified through IonGap Annotation Service. This sequence was compared also through BLAST to *B. licheniformis* 9945A ComX sequence with 100% identity. The amino acid sequence of standard 2 was based on the sequence of the recombinant plasmid.

The mobile phase of 20% acetonitrile in 0.1% aqueous ammonium acetate (w/v) at the flow rate of 1.0 ml min^−1^ washed the system for 5 min equilibration and continued to another 5 min after injection the sample into the column. The run was continued with a linear gradient of 20–55% acetonitrile in 0.1% aqueous ammonium acetate for 20 min. According to the retention time of the two standard samples, the associated fractions (standard 1 retention time: 14.5–15.5 min, and standard 2 retention time: 12.5–13.5 min) were collected and purified using Automated Fraction Collector Dionex UltiMate 3000 (Thermo Fisher Scientific). The pheromone molecule was detected at 210 nm. Detection of peptides and proteins in RP-HPLC, generally involves detection between 210 and 220 nm, which is specific for the peptide bond.

### Analytical methods

MS/MS and MALDI–MS used to determine the mass spectrometry and the amino acid sequences of pheromone peptide presented in the collected samples from HPLC. This work was performed by Proteomics Services at department of Biology, York University.

### Pheromone bioactivity assay

β-Galactosidase assay, using a *srfA*-*lacZ* reporter strain (*B. subtilis* JRL293), was performed according to the standard protocol (Tortosa et al. 2001). Different samples were tested to verify the bioactivity of pheromone presented in them. These samples are including; the supernatants which were obtained from the transformed *E. coli* BL21 cultures before and after the addition of IPTG as well as the supernatant from *B. licheniformis* NCIMB 8874 culture in the late exponential phase. Besides, the filtered extract from concentrated supernatant of induced recombinant *E. coli* BL21 (DE3) was also tested.

## Results

### Polymorphism of the quorum sensing locus at the protein level

To evaluate the presence of the *comQXPA* locus in the draft assembly of *B. licheniformis* protein, homologues of other bacilli were investigated after annotating the *B. licheniformis* NCIMB 8874 assembled sequence data through “IonGap Annotation Service” and aligning amino acid sequences of *comQXPA* cluster from this strain with homologous proteins from other Bacilli using “Clustal Omega” (Stark et al. 2010).

QS-related genes (*comQXPA*) previously identified and annotated in other Bacilli (Table 1) were compared to the *B. licheniformis* NCIMB 8874 genome (Accession number MBGK01000000) as homologues at protein level. The percentage identities for four annotated proteins (ComQ, ComX, ComP and ComA) are presented in Table 2.

**Table 1:** *Bacillus* species used for comparative analysis to QS-related genes in *B. licheniformis* NCIMB 8874

NCBI accession number	Organism	Competence	Genome annotation
NC_000964	*B. subtilis* subsp. 168	Competent	Annotated (Kobayashi et al. 2003)
NC_006270	*B. licheniformis* ATCC 14580	Non competent	Annotated (Rey et al. 2004)
GQ505081.1	*B. licheniformis* 9945A	Competent	*comQXPA*, *comS* and *mecA* annotated (Hoffmann et al. 2010)
GQ505080.1	*B. licheniformis* F11	Non competent	*comQXPA*, *comS* and *mecA* annotated (Hoffmann et al. 2010)
NC_009725	*B. amyloliquefaciens* FZB42	Competent	Annotated (Chen et al. 2007)
AF456135.1	*B. mojavensis* R-O-B2	Not identified	*comQXP* annotated (Ansaldi et al. 2002)
NZ_ACWC00000000	*Bacillus* sp. BT1B_CT2	Not identified	Annotated (unpublished)
NC_014479	*B. subtilis* subsp. *spizizenii* W23	Not identified	Annotated (unpublished)

**Table 2:** Percentage identities of ComQ, pre-ComX, ComP and ComA proteins of *B. licheniformis* NCIMB 8874 compared to homologues from other Bacilli

Organisms	ComQ			Pre-ComX			ComP			ComA		
	Accession No.	Length	%Identity	Accession No.	Length	%Identity	Accession No.	Length	%Identity	Accession No.	Length	%Identity
*B. licheniformis* 9945A	ADK89163.1	303	100	ADK89164.1	56	100	ADK89165.1	771	100	ADK89166.1	212	99
*B. licheniformis* 14580	Q65FH4	289	97	Q65FH5	54	38	Q65FH9	408	90	Q65FI0	212	99
*B. licheniformis* _F11	ADK89154.1	289	97	ADK89155.1	47	31	ADK89156.1	773	90	ADK89157.1	212	99
*B*. sp. BT1B CT2	EFV71237.1	293	93	EFV71236.1	57	57	EFV71235.1	766	93	EFV71234.1	212	99
*B. subtilis* subsp. *spizizenii* W23	ADM39121.1	286	52	AAL67740.1	58	30	ADM39119.1	774	66	ADM39118.1	214	79
*B. mojavensis* R-O-B2	AAL67730.1	286	51	AAL67731.1	54	48	–	–	–	–	–	–
*B. subtilis* 168	CAB07902.1	299	43	AAL67716.1	57	50	Q99027.3	769	67	P14204.1	214	79
*B. amyloliquefaciens* FZB42	ABS75210.1	286	40	ABS75209.1	57	40	A7Z882	766	65	ABS75207.1	214	77
*B. subtilis* R-o-FF1	–	–	–	AAL67716.1	57	50	–	–	–	–	–	–
*B. mojavensis* R-O-H1	–	–	–	AAF82177.1	53	49	–	–	–	–	–	–
*B. subtilis* R-O-E2	–	–	–	AAL67740.1	58	30	–	–	–	–	–	–
*B. mojavensis* R-O-C2	–	–	–	AAL67728.1	56	26	–	–	–	–	–	–
*B. subtilis* R-o-F3	–	–	–	AAL67737.1	73	22	–	–	–	–	–	–
*B. mojavensis*	–	–	–	–	–	–	ABB16431.1	559	62	–	–	–

In *B. licheniformis* NCIMB 8874, ComQ was identified as a 303-amino-acid protein. The alignment of *B. licheniformis* NCIMB 8874 ComQ with other homologues showed that the highest degree of identity appeared in other *B. licheniformis* strains such as 9945A, F11 and ATCC 14580. ComQ from *B. amyloliquefaciens* FZB42 appears to be the most divergent, with only 40% identity (Table 2).

**Fig. 1:**
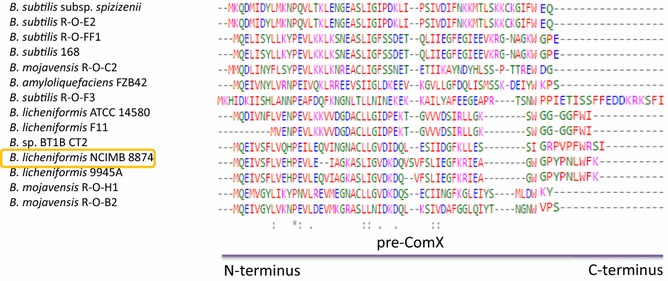
The amino acid sequence alignments of ComX precursor proteins across Bacilli listed in Table 2. Conservation appears restricted to the N-terminal protein ends. In contrast, high diversity in the C-terminus marks divergent within the pheromone-forming region

**Fig. 2:**
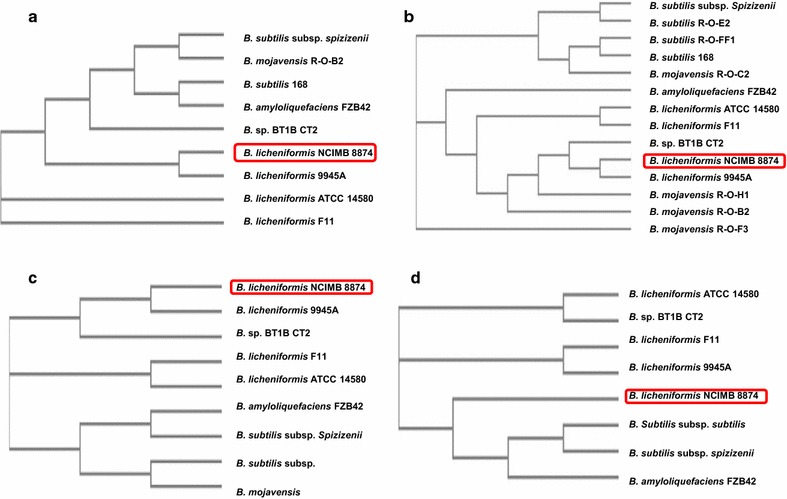
Phylogenetic tree analysis of the protein sequences of **a** ComQ, **b** pre-ComX, **c** ComP and **d** ComA using Clustal Omega. The *red box* denotes *B. licheniformis* NCIMB 8874. The distance values show the number of substitutions as a proportion of the length of the alignments

The precursor of ComX pheromone is a 56-amino-acid protein encoded within the *comX* locus in *B. licheniformis* NCIMB 8874 and according to these results, is highly polymorphic. Among *Bacillus* species, *B. licheniformis* 9945A and *B.* sp. BT1B CT2 share 100 and 57% identity, respectively. The other percentage identities range from 50 to 22 (Table 2). In pre-ComX, conservation appears restricted to the N-terminal protein ends. In contrast, high diversity in the C-terminus marks divergent within the pheromone-forming region (Fig. 1). Although the alignment of ComQ and pre-ComX sequences highlights the polymorphism of these proteins at the amino acid level, they could be classified into three main phylogenetic groups (Figs. 1, 2).

The *comP* nucleotide sequence of *B. licheniformis* NCIMB 8874 translated to a 771-amino-acid protein which performed as the sensor histidine kinase of the ComPA-two component system (Parkinson 1995; Kleerebezem et al. 1997; Bassler 1999, 2002). The results obtained from the amino acid sequence alignment revealed a variable distribution of identities ranging from 100% (*B. licheniformis* 9945A) to 62% (*B. mojavensis*) within ComP. Interestingly, polymorphism is restricted only to the N-terminal portion of the protein, whereas the C-terminus appears to be conserved (Additional file 1). Phylogenetic analysis further suggests, ComP homologues may be grouped in three distinct clusters (Fig. 2).

Conservation level for the 212-amino-acid protein ComA in Bacilli, confirmed this as the most conserved component of the QS-regulating cluster. Thus, the results showed 99% identity between *B. licheniformis* NCIMB 8874 and homologues from other *B. licheniformis* strains and *Bacillus sp.* BT1B_CT2. The lowest identity was observed with *B. amyloliquefaciens* FZB42 (Table 2). Phylogenetic analysis of ComA homologues at the protein sequence level again separated these into three main groups (Fig. 2). However, this phylogenetic tree shows the closest distance between *B. licheniformis* NCIMB 8874 and *B. subtilis* 168. It could be therefore postulated that the ComA functional implications across these two species might be similar.

### Purification and characterization of the ComX pheromone from *B. licheniformis* NCIMB 8874

**Fig. 3:**
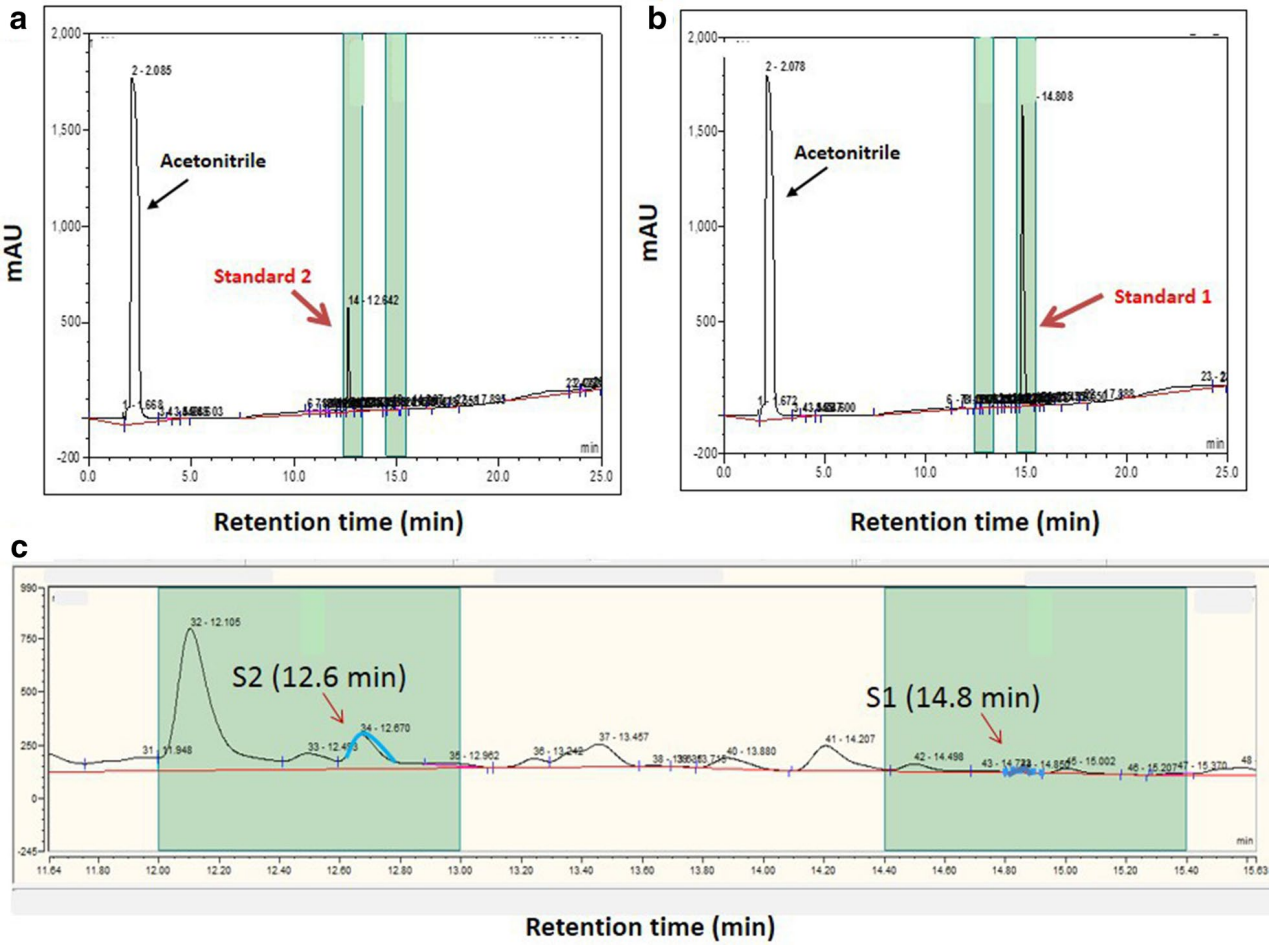
Chromatogram of standards and collected fractions associated to the standards retention times during gradient HPLC. **a** Standard 2 molecule (200 μg ml^−1^). **b** Standard 1 molecule (400 μg ml^−1^). **c** The zoomed fraction areas of one HPLC run related to extracted supernatant. Collection of the samples was performed from two fractions (presented in *green time* windows). First fraction was collected between 11.90 min and 12.90 min to cover the specific retention time of standard 2 (12.6 min). Second fraction was collected between 14.40 and 15.40 min according to the retention time of standard 1 (14.8 min). The *red arrows* presented in image b (S1 and S2) point at the interested peaks in the fractioning time which are representing the retention time of standard 1 and standard 2

*comQ* and *comX* were cloned under the control of a T7 promoter in the pET-22b(+) vector [pET-22b(+) *comQX*] and transformed into *E. coli* BL21 (DE3) which encodes T7 polymerase under the control of an IPTG-inducible promoter. To avoid contamination by medium components, defined media were used and an amino acid mix added to promote growth. Overproduced pheromone was recovered from the culture supernatant by reverse-phase chromatography and partial purification by gradient reverse-phase HPLC. To determine the optimal retention window for detecting the ComX pheromone, two peptides synthesised based on the *B. licheniformis* WGS data and the pET-22b(+) *comQX* plasmid sequence were used as pheromone standards (Table 3). Thus, two fractions were automatically collected by monitoring detection at 210 nm. Based on the retention time of the two standard samples, the associated fractions were collected and purified using the HPLC’s Automated Fraction Collector. The pheromone molecule was detected at 210 nm as this wavelength is used to measure active fractions (Fig. 3).

**Table 3:** Oligopeptide molecules used as standard samples in HPLC

Standards	Amino acid sequences	Molecular weight (gmol^−1^)	Retention time range (min)
Standard 1	WGPYPNLWFK	1307.5	14.5–15.5
Standard 2	KSWGGGGFWI	1094.2	12.5–13.5

**Fig. 4:**
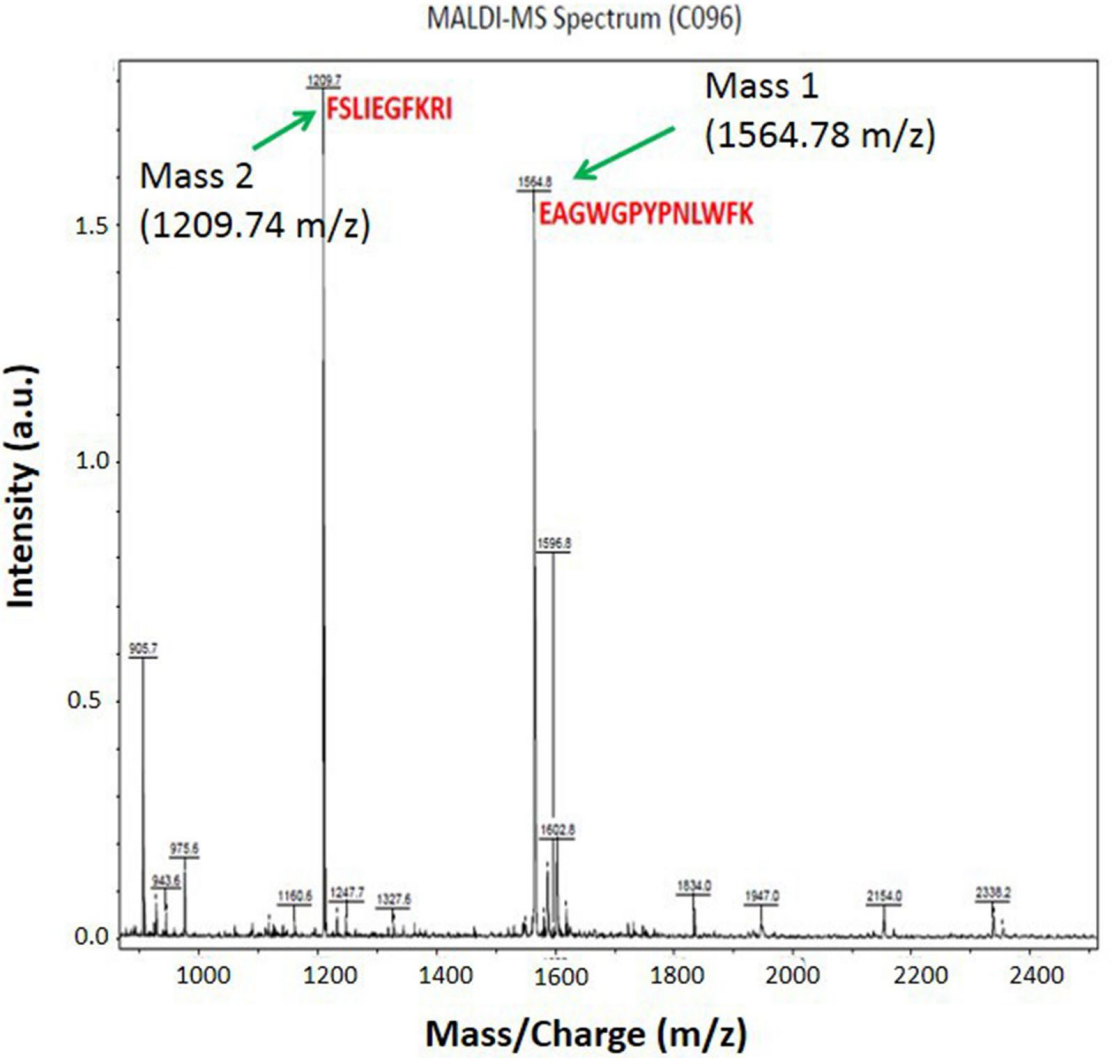
MALDI–MS spectra of the *B. licheniformis* QSM eluents obtained by HPLC with the two dominant peaks labelled with the resolved peptide sequence and analytically determined relative molecular mass

**Fig. 5:**
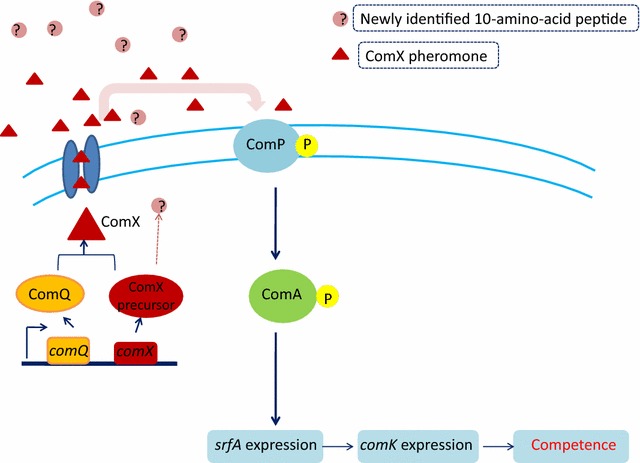
A schematic model for regulation of competence through extracellular signalling peptide-mediated QS in *B. licheniformis*. The ComX precursor is processed and secreted by ComQ into at least two separate peptides, one of which is the ComX pheromone. The transmembrane histidine kinase sensor protein (ComP) interacts with the ComX pheromone leading to phosphorylation of the ComA response regulator/transcription factor, which in turn drives transcription of the *srf* operon. The second peptide (marked ‘?’) has been identified in this study as a 10-amino-acid long component of the ComX precursor. The role of this new peptide is presently unknown

The fractions were next analysed by Tandem mass spectrometry (MS/MS) and Matrix-Assisted Laser Desorption/Ionization Mass Spectrometry **(**MALDI–MS), confidently (by the intensity of more than 1.5 a.u.) identifying two dominant ions (Fig. 4) as EAGWGPYPNLWFK (Mass 1) and FSLIEGFKRI (Mass 2). Mass 1 strongly matched the sequence of standard 1 (Table 3), whereas Mass 2 proved identical to the sequence of ComX precursor in *B. licheniformis* 9945A, as archived in UniProt, under the accession no. D9YRL0 (MQEIVSFLVEHPEVLEQVIAGKASLIGVDKDQVFSLIEGFKRIEAGWGPYPNLWFK). In the present study, this newly identified 10-amino-acid peptide has been reported for the first time, though its role has not yet been investigated in any QS system. Figure 5 shows a schematic model of competence regulation in *B. licheniformis* by presenting the role of the pheromone in QS system. The mature pheromone is generated by the processing and secretion of the precursor protein ComX by ComQ. Once exported in the extracellular environment the signalling molecule interacts with the histidine kinase sensor protein (ComP), triggering phosphorylation of the response regulator ComA. As a transcription factor, ComA binds to DNA and activates the transcription of the *srf* operon, thus inducing competence.

### Pheromone bioactivity

**Fig. 6:**
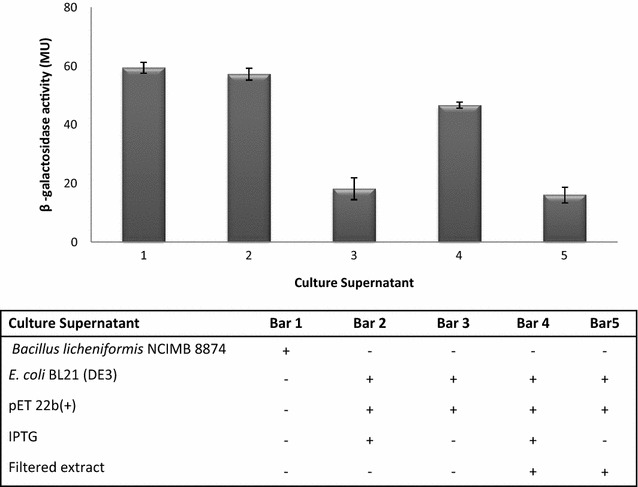
ComX bioactivity in bacterial culture supernatants from late exponential phase *B. licheniformis* NCIMB 8874 and *E. coli* BL21 ectopically expressing a *srfA*-*lacZ* fusion, as determined through a β-galactosidase reporter (*B. subtilis* JRL293). Data were collected from three independent experiments which were performed in triplicate. The constant blank (water) has been subtracted from all calculated values. The details of the tested culture supernatants are presented in the table

To explore ComX pheromone bioactivity a β-galactosidase reporter assay was used based on a reporter, *B. subtilis* JRL293 strain, carrying a *srfA*-*lacZ* fusion enabling pheromone activity quantification through β-galactosidase activity induction (Fig. 6). These studies demonstrated that both *B.licheniformis* NCIMB 8874 in the late exponential growth phase and *E. coli* BL21 (DE3) carrying the IPTG-induced ComQX expression cassette in pET-22b(+) resulted in comparable levels of supernatant ComX bioactivity, and therefore comparable pheromone yields. Moreover, filtration extraction resulted in minor ComX activity loss.

## Discussion

The proteins encoded in *comQXPA* have been investigated and compared with homologues in related species. These studies have shown that the competence regulating locus is highly polymorphic across ComQ, ComX and the region of ComP encoding the N-terminal part of the protein, whilst the C-termini of ComP and ComA are highly conserved (Tran et al. 2000; Tortosa et al. 2001). Our own analysis performed on *comQXPA* locus of *B. licheniformis* NCIMB 8874 as sequenced in the present study confirmed further evidences for this pattern of molecular evolution since the ComQ and ComX coding regions of strains WX-02 and NCIMB 8874 were found to share only 85% identity at the nucleotide level. However these regions in ATCC 14580 strain showed 93% identity with NCIMB 8874 strain (Lapidus et al. 2002).

Previous research has revealed that the *Bacillus* pheromones can be classified in four pherotypes depending on their amino acid sequences and the nature of the post-translational modifications on their tryptophan residues. Accordingly, the pheromones belonging to the same group are able to generate a cross-induction phenomenon (Ansaldi et al. 2002). In this context, the confirmed polymorphism in the *comQXP* locus suggests a striking pattern of specificity in pheromone interactions with the receptor protein ComP (Tran et al. 2000; Tortosa et al. 2001). However, the present study demonstrated that the ComX pheromone generated by *B. licheniformis* NCIMB 8874 was able to activate a QS response in *B. subtilis*. Therefore, comparative analysis between the *comQXPA* loci of NCIMB 8874 strain and other selected Bacilli supported the further investigation of the relationships between the polymorphisms in this cluster and the specificity exerted in the QS system across different *Bacillus* spp. and strains.

The utility of reporter systems in the study of bacterial inter-species communication has been demonstrated with respect to *the B. subtilis* AI-2 signal against low cell-density *Vibrio harveyi*, a Gram-negative bacterium (Lombardia et al. 2006). In the current study the reporter strain was used to monitor the expression of the *srfA* operon which is a known requirement for competence development. Thus, *B. subtilis srfA*-*lacZ* cultures at low cell densities show *srfA* expression at basal level. Accordingly, the level of β-galactosidase activity indicates *srfA* expression levels induced by signalling molecules accumulated in the extracellular medium (Magnuson et al. 1994). The bioassay showed that the supernatant of *B. licheniformis* NCIMB-8874 collected at the late exponential growth phase as well as IPTG-induced supernatant from *E. coli* cells transformed with the *comQX* cassette resulted in the highest expression *lacZ* pointing towards a comparably high pheromone bioactivity in both samples. The small activity reduction observed after filtration could be attributed to the partial inactivation or loss of the pheromone during purification. In contrast, in the absence of IPTG the transformed *E. coli* cells show 39 Miller Unit (MU) less pheromone activity, compared to the induced supernatant as a result of *srfA* expression at the basal level. Future studies should explore the use of *B. licheniformis* reporter strains to improve the accuracy of data acquisition. Such constructs would contribute to better elucidate QS-regulated secondary metabolite production in the less well known system of *B. licheniformis*.

To this end, the products of the *comQXP* locus were individually aligned with homologues, *B. licheniformis* strains were usually found in the same cluster, alongside the less well-described *Bacillus* sp. BT1B_CT2. Interestingly, the cluster components ComQ, ComX precursor and ComP across two strains of *B. licheniformis*, ATCC 14580 and F11, were classified under a distinct group. Previously, it has been reported that these two strains of *B. licheniformis* harboured non-functional QS systems (Hoffmann et al. 2010). ComA congruence indicated that, whilst this protein is conserved in the same bacterial species, the conservation does not extend to the genus. These findings bear interesting implications on the putative conservation of ComA.

Although *B. subtilis* subsp. *subtilis* and *B. licheniformis* NCIMB 8874 were classified under different phylogenetic groups for ComX and ComP, their positions on pre-ComX evolutionary tree are not too distant, thus confirming the possibility of cross induction between the two species. This is experimentally supported by the pheromone bioactivity outcomes of our reporter studies. The amino acid sequence alignments between the two ComX precursor proteins, however, showed that their conservation is only restricted to the N-terminal ends. Moreover, high diversity in the amino acid sequence in C-terminus marked the pheromone-forming region, where, interestingly, the tryptophan residue is located. Classification of this pheromone under a particular pherotype based on amino acid sequence is not possible since little is known about the mechanism of its modification by ComQ. As our experimental evidence suggests that the ComX pheromone of *B. licheniformis* NCIMB 8874 may induce a QS response in a *B. subtilis* reporter strain derived from *B. subtilis* subsp. *subtilis*, despite the obvious amino acid sequence divergence. We postulate a common tryptophan modification might account for the observed functional overlap. Interestingly, the percentage identities of pre-ComX and ComP proteins of *B. licheniformis* NCIMB 8874 and other Bacilli showed that the similarity of pre-ComX of *B. licheniformis* NCIMB 8874 to associated protein in *B. mojavensis* and *B. subtilis* strains is 20–50% while the identity in ComP is 60–70%. This could be an evidence for the existence of amino acid sequence simillarity in ComP than pre-ComX.

Furthermore, null mutants of *comQ* fail to mature the ComX precursor into a bioactive pheromone (Magnuson et al. 1994). Indeed Tortosa and colleagues reported that co-expression of *comQ* and *comX* in *E. coli* leads to the production of active pheromone in the medium, demonstrating that ComQ is the only dedicated protein required for processing, modification, and release of active ComX pheromone (Tortosa et al. 2001). Our present studies are therefore focusing on the nature of tryptophan modifications present, if any.

In the current study, the finding about the importance of the co-expression of *comQ* and *comX* for pheromone production in *B. licheniformis* NCIMB 8874, helped to design a suitable primer set and subsequently conduct successful *comQX* gene cloning to produce ComX pheromone of *B. licheniformis* NCIMB 8874. Applying the plasmid pET-22b(+) as the vector, and the restriction enzymes (*Bam*HI and *Nde*I) introduced in *B. subtilis* QS studies (see the methods in Schneider et al. 2002; Ansaldi et al. 2002), led to successful cloning of *comQX* genes of *B. licheniformis* NCIMB-8874 for the first time in the present research. The cloned *comQX* genes was also sequenced and verified in the BLAST algorithm.

The recent investigation of *comQXPA*-like genes in 2620 complete/6970 draft prokaryotic genomes shows that in addition to *B. subtilis* and its close relatives, 20 *comQXPA*-like loci are identified outside the *B. subtilis* clade, all in the phylum Firmicutes. The sequence variability in the ComX peptide is evident in both *B. subtilis* and non-*B. subtilis* clade which suggests grossly similar evolutionary constraints in the underlying quorum sensing system (Dogsa et al. 2014). The pre-ComX protein sequence comparison analysis between *B. licheniformis* and other Bacilli lead to similar evolutionary conclusion as Dogsa et al. studies since the percentage identity varied from 20 to 100%.

On the basis of these functional observations, we further studied the *B. licheniformis* QS system components produced recombinantly in *E. coli.* Mass spectrometry matched one of the two main peaks (Mass 1, 13 amino acid peptide) to the *B. licheniformis* NCIMB-8874 ComX precursor C-terminus, a synthetic analogue of which we had used as an assay standard (standard 1). Therefore, this sequence most likely corresponds to the ComX pheromone sequence as it compares well with the historically described *B. subtilis* ComX (Schneider et al. 2002). Curiously, the last reported QS peptide from *B. subtilis* Ro-E-2 features a distinctly different, 6-amino-acid residue sequence: GIFWEQ (Okada et al. 2005). Given the nucleotide level identity between *B. licheniformis* NCIMB-8874 pre-*comX* and *B. subtilis* Ro-E-2 is only 31%, we postulate that the ComX pheromone amino acid residue in these two strains varies substantially both in size and sequence. This is supported by the degree of phylogenetic divergence across bacilli as reported herein. Moreover, the *B. licheniformis* NCIMB-8874 strain pheromone shows a N-terminal cleavage site substantially different to that characterised by Ansaldi and co-workers in *B. subtilis* 168 (2002); thus mature peptides in the Ansaldi et al. studies exhibited diverse lengths ranging 5–10 amino acids.

Interestingly, the second sequence (Mass 2, 10-amino-acid peptide) aligns well with the element of the precursor ComX molecule found in *B. licheniformis*. This is the first report suggesting that elements of the ComX precursor are also secreted from these bacteria along with the bioactive pheromone peptide. It is presently unclear if this fragment has any biological significance within host QS or the communication systems of other bacteria (Fig. 4).

In conclusion, this project has utilised whole genome sequencing to identify the QS locus in *B. licheniformis,* enabling its sub-cloning and the biochemical production and characterisation of the previously undescribed *B. licheniformis* ComX pheromone. Further studies on the chemical structure of this cell communication compound are warranted in the future work alongside the elucidation of any antimicrobial role of the pheromone itself, or the newly described, co-produced precursor fragment (ComE coming from Com-Elham).

## Additional file

**Additional file 1: Figure S1.** Clustal Omega multiple sequence alignment of ComP. Conserved amino acids are indicated with the same colours in all rows.

## Abbreviations

PGM: personal genome machine
NGS: next generation sequencing
WGS: whole genome sequencing
QS: quorum sensing
QSMs: quorum sensing molecules
