# Supplementary material for: Genomic and molecular characterization of a novel quorum sensing molecule in Bacillus licheniformis
Source: AMB Express. 2017 Apr 8;7:78. doi: 10.1186/s13568-017-0381-6 (PMC5385187; doi:10.1186/s13568-017-0381-6)

**Figure S1** Clustal Omega multiple sequence alignment of ComP. Conserved amino acids are indicated with the same colours in all rows.


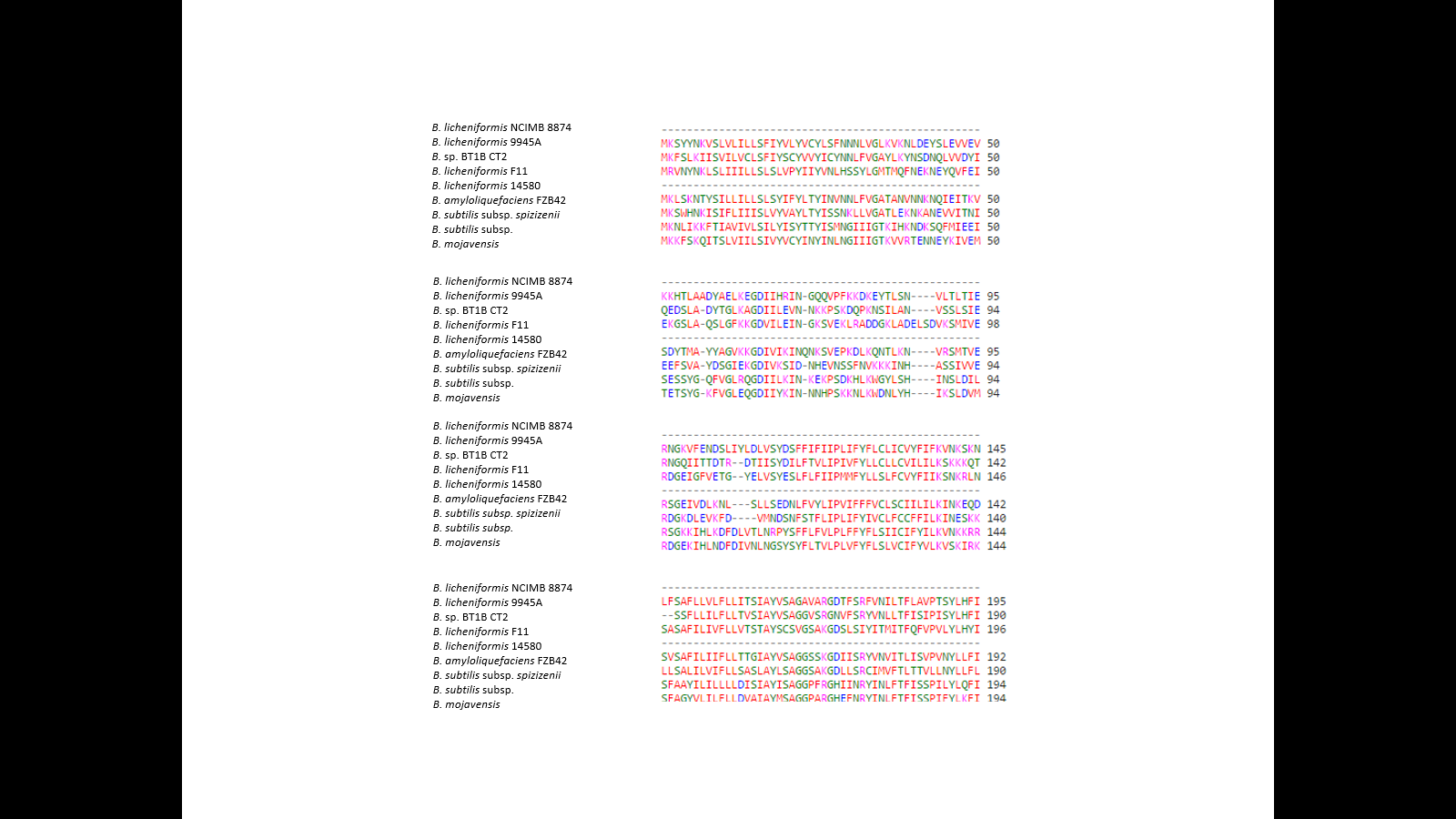


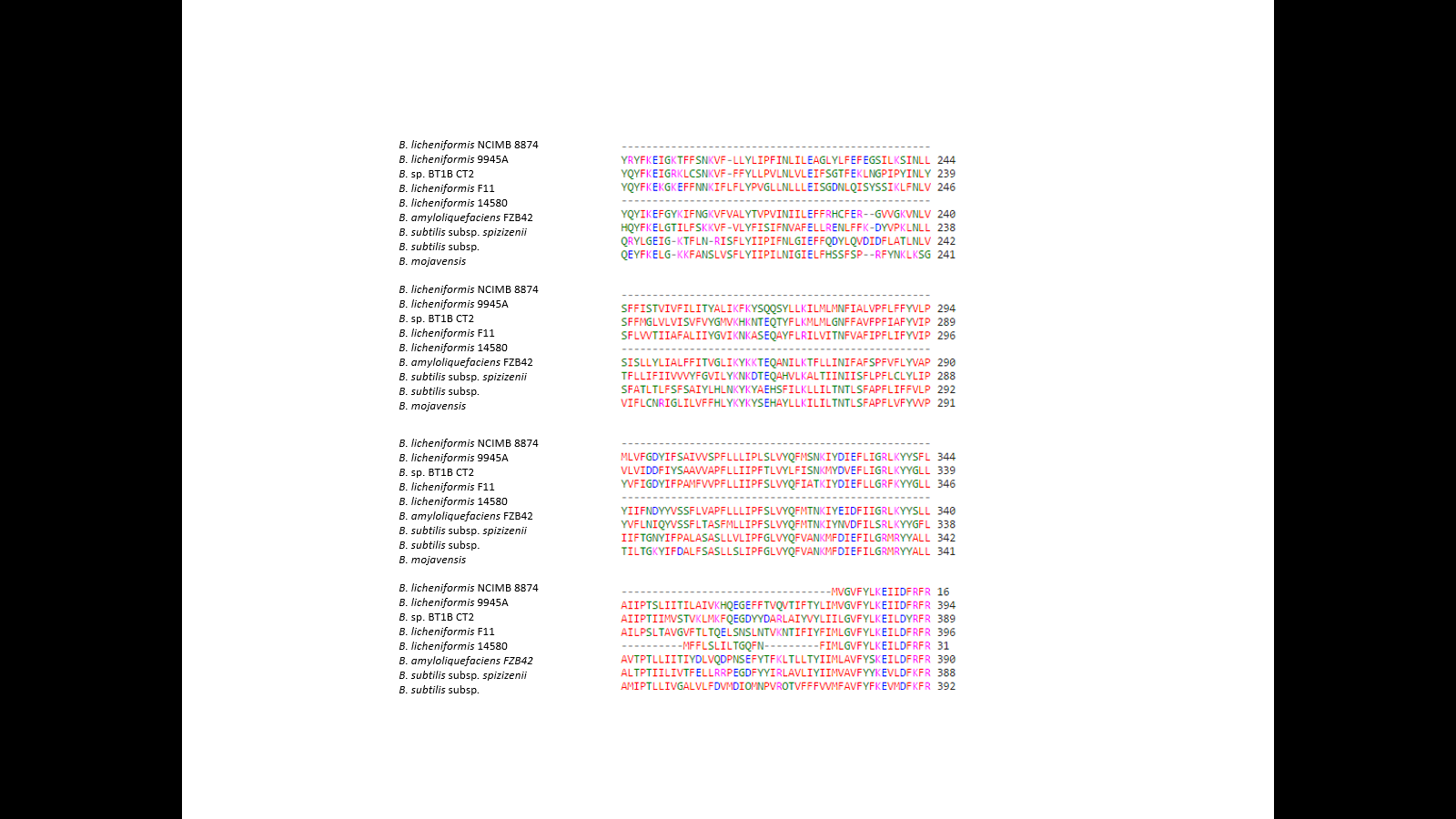


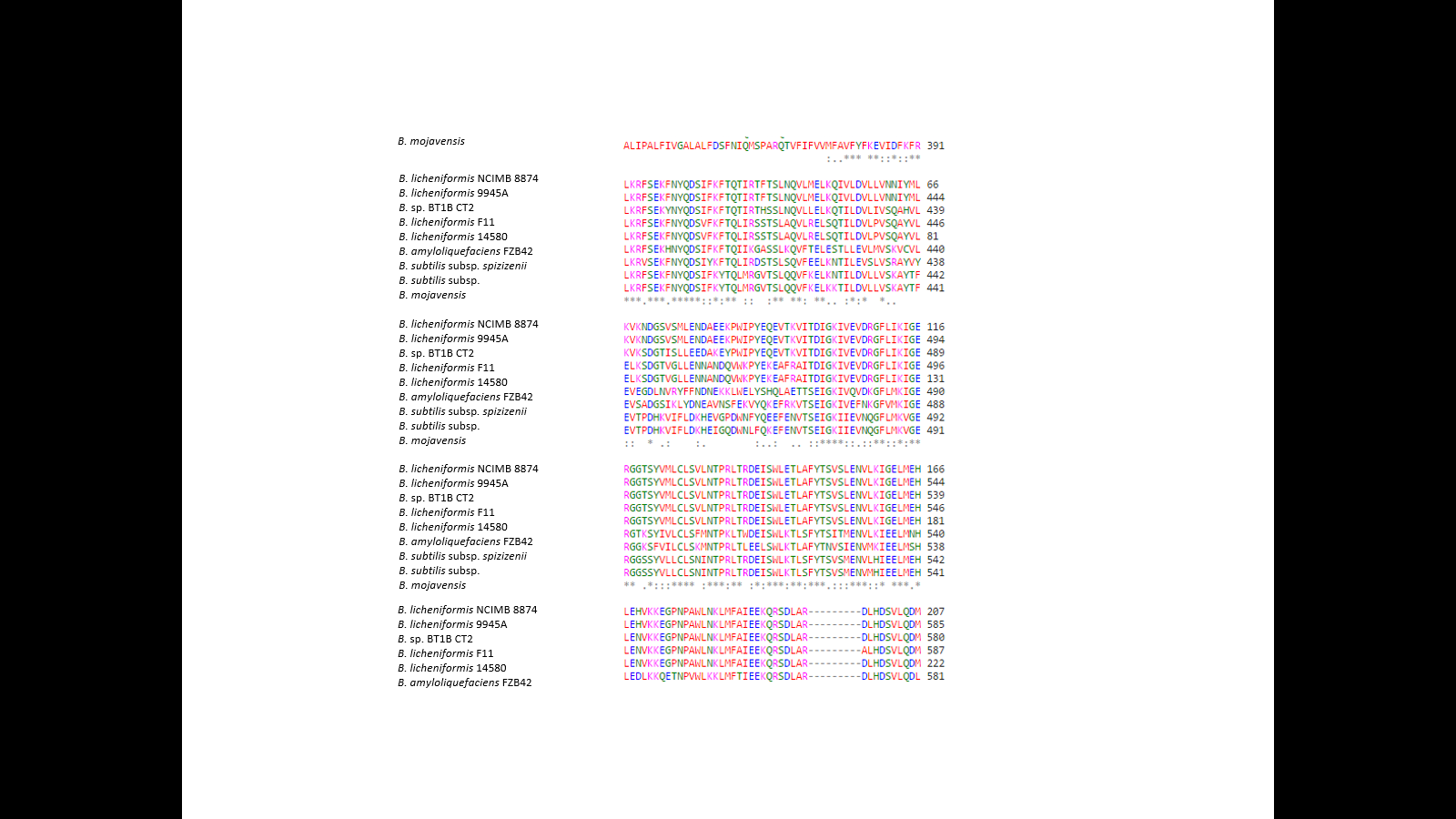


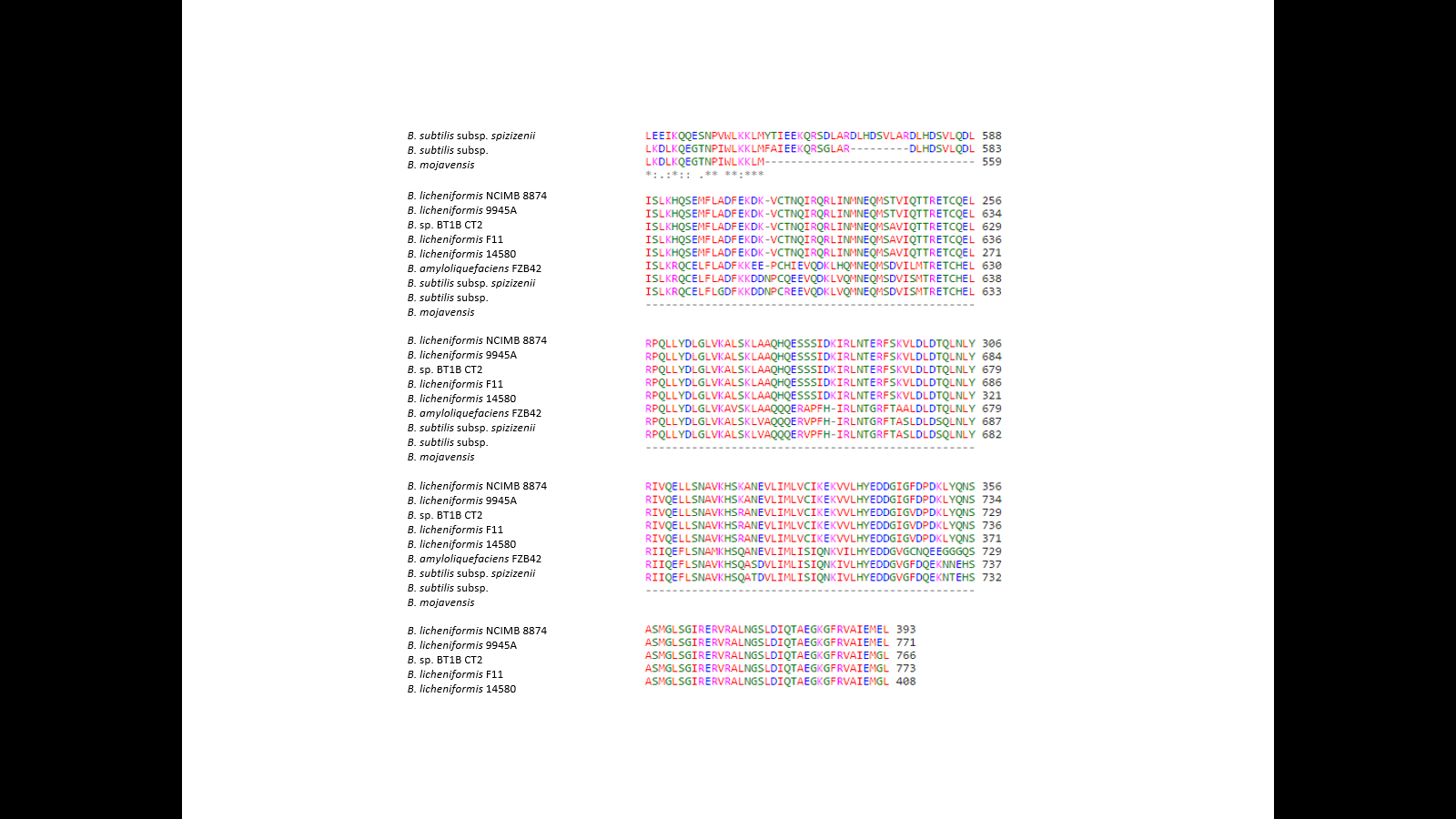


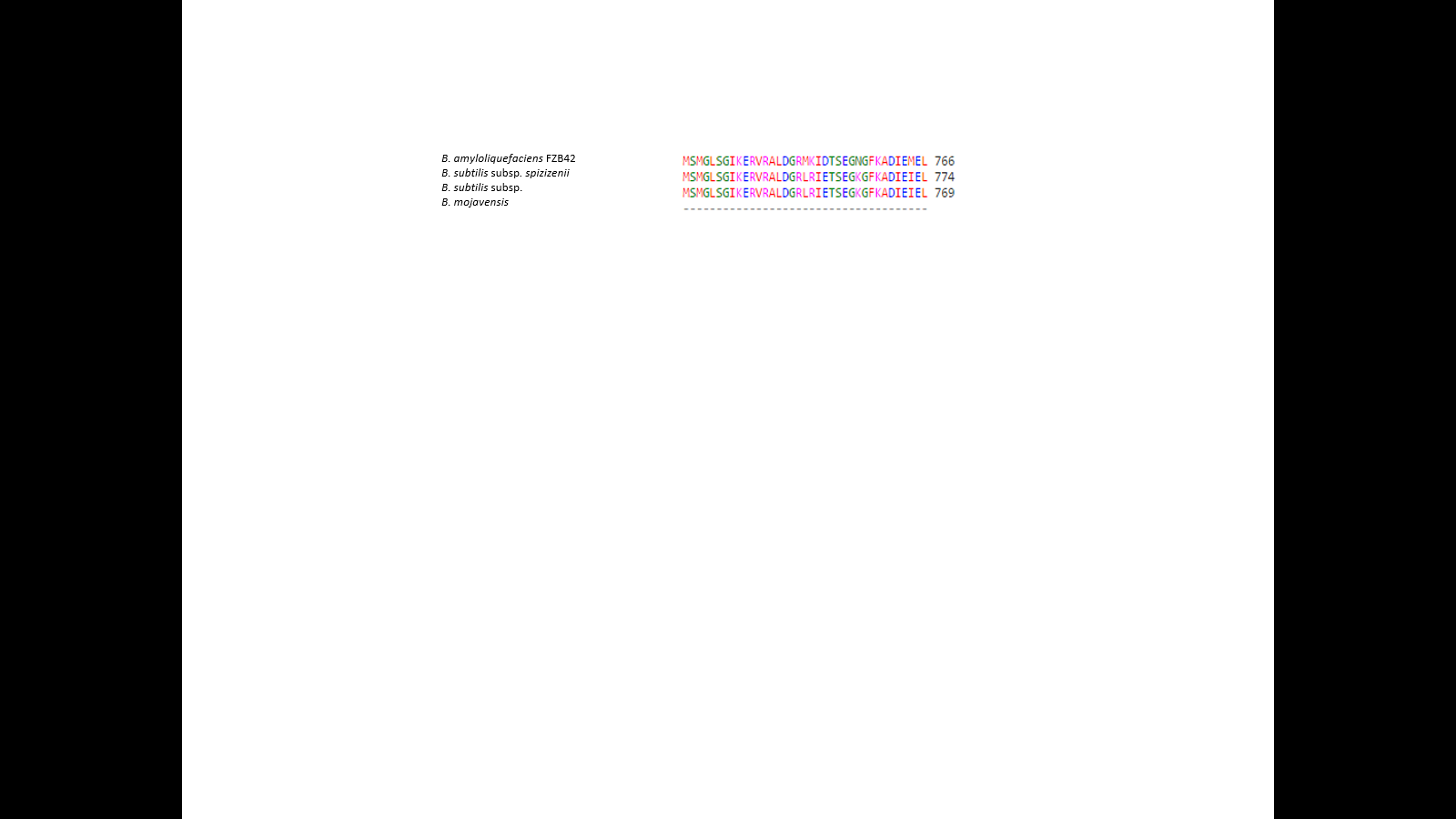

Supplement: Supplementary file 1 — Additional file 1: Figure S1. Clustal Omega multiple sequence alignment of ComP. Conserved amino acids are indicated with the same colours in all rows. [file 13568_2017_381_MOESM1_ESM.docx]
